# Linear IgA bullous dermatosis: 32 years of experience^☆^

**DOI:** 10.1016/j.abd.2024.03.015

**Published:** 2024-12-10

**Authors:** Tugba Atci, Gizem Pehlivan Ulutas, Ecem Güreler Sirkeci, Rıfkiye Küçükoğlu

**Affiliations:** a Department of Dermatology and Venereology, Istanbul Medical Faculty, Istanbul University, Istanbul, Turkey; b Sultangazi Haseki Training and Research Hospital, Istanbul, Turkey

**Keywords:** Linear IgA bullous dermatosis, Autoimmune diseases, Skin diseases, Vesiculobullous

## Abstract

**Background:**

Linear IgA bullous dermatosis (LABD) is an uncommon disease with only a few reported studies in large series with long follow-up periods.

**Objectives:**

To evaluate the clinical presentation, immunopathological features, management, and disease course in LABD patients.

**Methods:**

Data including demographics, clinical features, histopathological and immunofluorescence findings of LABD patients, in addition to the preferred treatments and responses to treatments were evaluated.

**Results:**

Among 26 patients diagnosed with LABD, 17 (65.4%) were female. The mean age was 40.3 ± 22.4 (6‒80) years of whom 21 were adults. The most common mucosal involvement was oral (n = 9, 34.6%). Continuous linear IgA deposition was present on the basement membrane zone of all patients in addition to C3 (n = 13), IgG (n = 9), IgM (n = 4), and fibrinogen (n = 4). Three patients were lost to follow-up without any treatment. Dapsone was the treatment of choice in most (n = 21, 91.3%) patients in addition to systemic corticosteroids (n = 17), azathioprine (n = 3), tetracycline and nicotinamide (n = 2). Complete and partial remissions were achieved in 11 (47.8%) and 12 (52.2%) patients, respectively, in a mean follow-up period of 45.9 ± 43.9 (3‒158) months. Furthermore, 17 patients were still under treatment at the end of the follow-up period.

**Study limitations:**

Retrospective study conducted in a single center.

**Conclusions:**

LABD may occur at two separate peaks, one in the second and the other in the sixth decade of life with a female predominance. Other immunoglobulins may be associated with dominant IgA antibody deposition and the most commonly used therapeutic option for LABD patients was oral dapsone.

## Introduction

Linear IgA bullous dermatosis (LABD) is an uncommon acquired autoimmune subepidermal vesiculobullous disease that is characterized by continuous linear IgA deposits on the basement membrane zone visualized on direct immunofluorescence microscopy (DIF).^1^, ^2^, ^3^, ^4^, ^5^ Compared with other immunobullous diseases, LABD is immunologically a heterogeneous disease with pathogenic IgA autoantibodies against different hemidesmosome antigens.^2^, ^6^ The disease affects adults and children with only a few reported studies on large series in the literature.^1^, ^2^, ^3^, ^4^, ^5^, ^6^, ^7^, ^8^, ^9^, ^10^, ^11^, ^12^, ^13^, ^14^, ^15^, ^16^, ^17^, ^18^, ^19^, ^20^, ^21^ Given the rarity of LABD, its definition, long-term follow-up, and the natural course of the disease are still not fully elucidated.^1^, ^2^, ^3^, ^4^, ^5^, ^6^, ^7^, ^8^, ^9^, ^10^, ^11^, ^12^, ^13^, ^14^, ^15^, ^16^, ^17^, ^18^, ^19^, ^20^, ^21^, ^22^ The authors aimed to evaluate the clinical presentation, immunopathological features, management, and disease course in the patients diagnosed with LABD with a long-term follow-up and compare these findings with literature data.

## Materials and methods

This is a retrospective study of all consecutive patients diagnosed with LABD between 1990 and 2022 in a tertiary dermatology center. The diagnosis of LABD was confirmed by histopathological and immunofluorescence (direct and indirect) findings, in addition to Enzyme-Linked Immunosorbent Assay (ELISA) levels of anti-BP180 and/or anti-BP230 antibodies. Histopathological examination of all patients showed subepidermal bullae with neutrophilic and/or eosinophilic infiltrates in the papillary dermis. The DIF examination of perilesional skin showed isolated linear IgA deposition or dominant linear IgA deposition in addition to concomitant linear IgG, IgM, and/or C3 deposition in all patients. The data including gender, age at diagnosis, clinical, histopathological and immunofluorescence findings were obtained from a review of patients' medical records. In addition, comorbidities, possible triggering factors, the choice of treatments, and outcomes at the end of follow-up time were also recorded. The patients’ quantitative data are expressed as a number, percentage, mean, or median (range).

The patients in this manuscript have given written informed consent to the publication of their case details. This study was conducted in accordance with the ethical standards of the institutional committee and with the Declaration of Helsinki (Approval number: E-29624016-050.99-816593).

## Results

Among a total of 984 patients followed up at our autoimmune bullous disease outpatient clinic, only 26 (2.6%) were diagnosed with LABD in the last 32 years. The mean age of LABD patients at the time of diagnosis was 40.3 ± 22.4 (6‒80) years, of whom 17 (65.4%) were female. Most of them (n = 21) were adults with a mean age of 47.2 ± 18.9 years and five were children with a mean age of 11 ± 4.9 years at the time of diagnosis. In addition, LABD most commonly occurred in the second (n = 4, 15.4%) and sixth decade (n = 7, 26.9%) in these patients (Table 1).

**Table 1:** Demographic characteristics, comorbidities and possible triggering factors of patients with linear IgA bullous dermatosis.

Characteristics	n (%)
**Demographics**	n = 26
**Gender**	
Male	9 (34.6)
Female	17 (65.4)
**Age group at onset of LABD (years)**	n = 26
0‒9	3 (11.5)
10‒19	4 (15.4)
20‒29	3 (11.5)
30‒39	3 (11.5)
40‒49	1 (3.8)
50‒59	7 (26.9)
60‒69	3 (11.5)
70‒79	1 (3.8)
80‒89	1 (3.8)
**Comorbidities**	n = 26
Hypertension	3 (11.5)
Diabetes mellitus	3 (11.5)
Congestive heart failure	3 (11.5)
Osteoporosis	2 (7.7)
Epilepsy	2 (7.7)
Hyperlipidemia	2 (7.7)
Cirrhosis	2 (7.7)
Hypothyroidism	2 (7.7)
Others	8 (30.8)
**Possible triggering factors**	n = 26
Pregnancy	2 (7.7)
Lung carcinoma	1 (3.8)
Infection/antibiotherapy	1 (3.8)

Hypertension (n = 3), diabetes mellitus (n = 3), congestive heart failure (n = 3), osteoporosis (n = 2), epilepsy (n = 2), hyperlipidemia (n = 2), cirrhosis (n = 2), and hypothyroidism (n = 2) were most commonly associated comorbidities in adult patients (Table 1). Chronic renal failure, diabetes insipidus, essential thrombocytosis, asthma, chronic obstructive pulmonary disease, rheumatoid arthritis, gastritis, and myocardial infarction were other comorbidities that were recorded in one adult patient each. The possible triggering factors for disease onset found in patients were pregnancy (n = 2), lung carcinoma (n = 1), urinary tract infection and antibiotherapy (n = 1) (Table 1).

**Figure 1:**
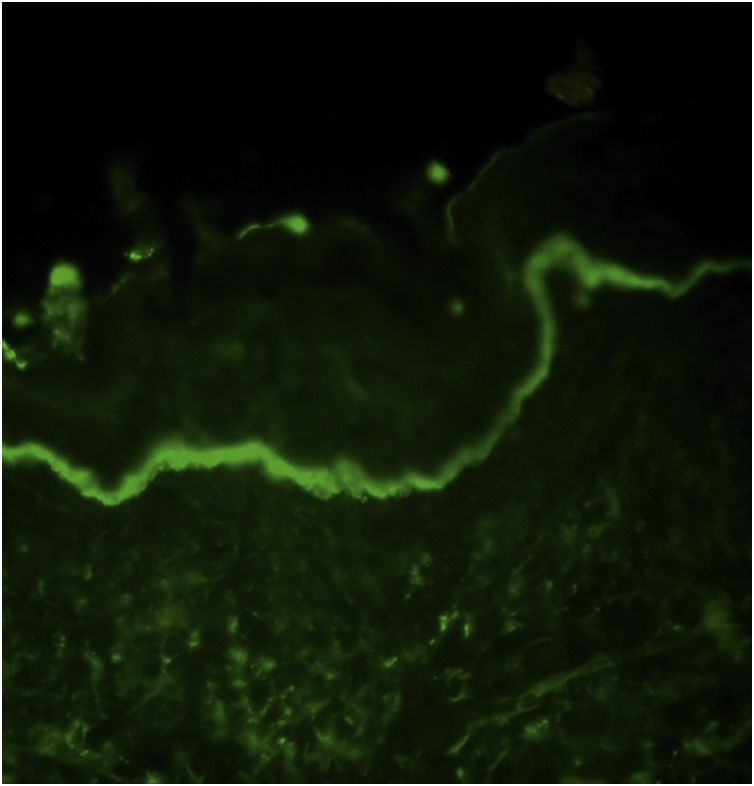
The direct immunofluorescence examination demonstrates a continous linear IgA deposition along the basement membrane zone in a patient with linear IgA bullous dermatosis.

In DIF examination, continuous linear IgA deposition was present on the basement membrane zone of all patients (Fig. 1) in addition to C3 (n = 13), IgG (n = 9), IgM (n = 4), and fibrinogen (n = 4) (Table 2). The authors could perform Indirect Immunofluorescence (IIF) in 18 patients of whom six showed IgA deposition at the basement membrane zone. In addition, two of them had IgG positivity at the basement membrane zone, as well. Anti-BP230 IgG antibodies were detected positive with ELISA in only two patients (Table 2).

**Table 2:** Clinical and laboratory characteristics of patients with linear IgA bullous dermatosis.

Characteristics	n (%)
**Clinical Manifestation**	n = 26
Only skin lesion	17 (65.4)
Skin and mucosal lesions	9 (34.6)
-Oral lesion	9 (34.6)
-Ocular lesion	1 (3.8)
-Nasal lesion	1 (3.8)
**DIF findings**	n = 26
IgA ± IgM,C3	17 (65.4)
IgA + IgG ± IgM,C3	9 (34.6)
**IIF findings**	n = 26
IgA+, IgG+	2 (7.7)
IgA+, IgG-	4 (15.4)
IgA-, IgG-	12 (46.2)
NA	8 (30.8)
**Serological (ELISA) findings**	n = 26
BP180/230 IgG-	9 (34.6)
BP230 IgG+	2 (7.7)
Bp180 IgG+	0 (0)
NA	15 (57.7)

DIF, Direct Immunofluorescence; IIF, Indirect Immunofluorescence; NA, Non-Available.

**Figure 2:**
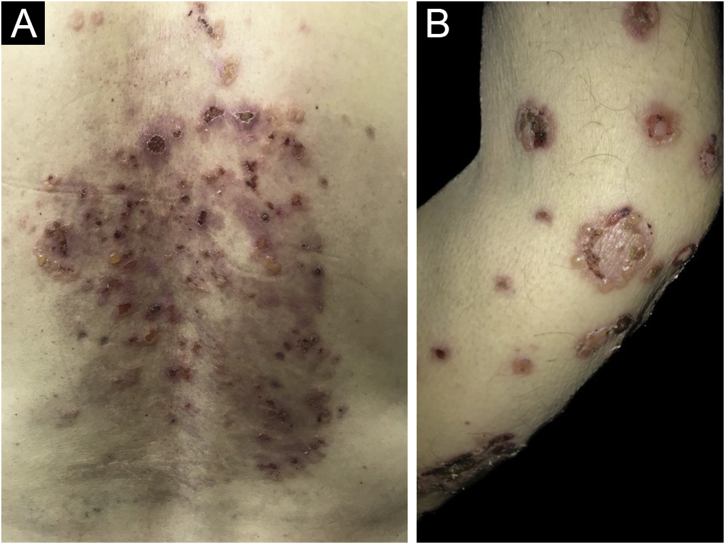
(A) Vesiculobullous skin lesions with erosion and crusting limited to the back of an adult patient with linear IgA bullous dermatosis. (B) Arciform vesiculobullous lesions in a “cluster of jewels” configuration in an adult patient with linear IgA bullous dermatosis.

**Figure 3:**
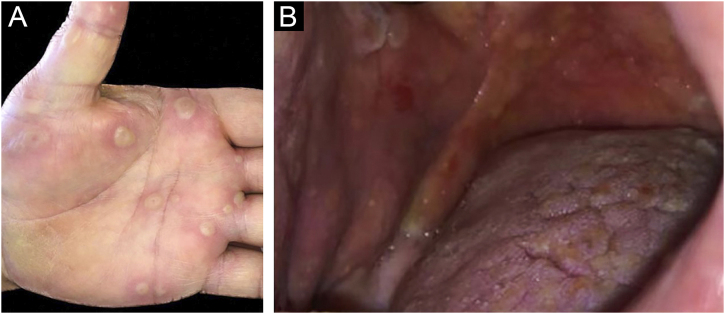
(A) Tense vesiculobullous lesions in the palmar region in an adult patient with linear IgA bullous dermatosis. (B) Erosions and tense bullous lesions on the oral mucosa of an adult patient with linear IgA bullous dermatosis.

Clinically, vesiculobullous skin lesions with erosion and crusting sometimes with an annular appearance were seen in all LABD patients (Figs. 2 and 3 A). While in 17 (65.4%) patients the disease presented with skin lesions alone, in others (n = 9, 34.6%) mucosal involvement was associated with skin lesions. The most common mucosal involvement was oral (n = 9) (Fig. 3B), followed by ocular (n = 1) and nasal mucosa (n = 1), all of which were recorded in adult patients with LABD (Table 2).

Three patients were lost to follow-up without any treatment and other (n = 23) patients were followed up for a mean period of 45.9 ± 43.9 (3‒158) months. Dapsone was the treatment of choice in most (n = 21, 91.3%) patients in addition to systemic corticosteroids (SC) (n = 17), azathioprine (n = 3), tetracycline and nicotinamide (n = 2). Complete and partial remissions were achieved in 11 (47.8%) and 12 (52.2%) patients, respectively, and 17 patients were still under treatment at the end of the follow-up period (Table 3). In all child patients with LABD, dapsone was utilized at a dosage ranging from 0.5 to 1 mg/kg per day. Additionally, two of these patients received SC treatment in conjunction with dapsone therapy. One was lost to follow-up and the other four children had a median follow-up duration of 21 (2‒129) months with a partial treatment response in three of them.

**Table 3:** Treatments and treatment response of patients with linear IgA bullous dermatosis.

Characteristics	n (%)
**Treatment**	n = 23
Dapsone	21 (91.3)
Systemic corticosteroid	17 (73.9)
Azathioprine	3 (13)
Nicotinamide and tetracycline	2 (8.7)
Colchicum	1 (4.3)
Topical corticosteroid/tacrolimus	1 (4.3)
Other (Mycophenolate mofetil, cyclophosphamide, immunoadsorption, intravenous immunoglobulin, rituximab, cyclosporine A)	1 (4.3)
**Treatment response**	n = 23
Treatment discontinuation due to complete remission	6 (26.1)
Complete remission under treatment	5 (21.7)
Partial remission under treatment	12 (52.2)

## Discussion

LABD is a rare autoimmune subepidermal bullous disease with an insufficiently understood pathogenesis. LABD has distinct characteristics in adults and children, in terms of potential triggers, clinical manifestations, and prognosis with only a few studies on large series. In addition, the long-term evolution of the disease is poorly described in most of them.^1^, ^3^, ^4^, ^7^, ^8^, ^9^, ^10^, ^11^, ^12^, ^13^ In this retrospective study, the authors evaluated the clinical characteristics, treatments and outcomes of 26 patients of LABD with a relatively long follow-up period; the first large case series reported from Turkey.

LABD is not only a clinically heterogeneous disease but also one that produces heterogeneous autoantibodies targeting multiple antigens.^2^, ^8^ The 97 and 120 kd are cleavage products of Bullous Pemphigoid antigen 2 (BP180) and are major antigenic targets for IgA autoantibodies.^1^ Other autoantigens described are BP230, type VII collagen, and laminin 332.^5^, ^11^, ^14^ Anti-BP230 IgG antibodies were also detected positive with ELISA in our two patients (Table 2).

LABD may occur at two separate peaks, one in the teenage and early adult years and the other in the sixth decade of life with a female predominance, as in the studied patients (Table 1).^2^ In children, the age of onset is classically in the preschool years which is also known as chronic bullous dermatosis of childhood, the most common autoimmune bullous dermatosis found in children.^3^, ^6^, ^12^ In contrast to adults, males may be more commonly affected by childhood LABD, as in this series (M/F: 1.5).^12^ The two variants have certain clinical differences, but their histologic and DIF findings are identical.^2^, ^6^

The onset of LABD is frequently preceded by one of several triggering factors, including drugs, vaccinations, infections, and autoimmune disorders.^3^, ^4^, ^15^ Often LABD occurs in the setting of a coexisting infection or underlying disease, which is thought to act as an immunological trigger in the development of bullous disease, as seen in one of the patients. This raises the question of the role of the underlying infectious disease itself or the role of only the drug itself.^2^ Vancomycin has been suspected in more than half of the cases of drug-induced LABD patients which was not recorded in the studied patients.^14^ Previously, it was shown that drug-induced LABD may show spontaneous remission following cessation of the responsible drug. However, in some patients drug-induced LABD may be more severe than idiopathic LABD mimicking toxic epidermal necrolysis.^6^, ^14^

Although the association of ulcerative colitis with LABD has been reported previously in approximately 7% of patients, this association was not recorded in the present series.^1^, ^4^, ^5^, ^10^^,^^13^, ^16^, ^17^, ^18^, ^19^ Among those with such an association, ulcerative colitis preceded the onset of LABD in 94% of the patients and interestingly colectomy improved LABD lesions in some patients.^5^, ^18^ It has been hypothesized that intestinal inflammation may induce the exposure and presentation of intestinal antigens including BP180 that are cross-reactive to cutaneous antigens, stimulating autoimmune response to antigens of cutaneous basement membrane zones.^4^, ^10^, ^18^ Future studies may clarify the causal relationship between these two diseases. Although adult LABD patients had many comorbidities, a striking association of any comorbidity with LABD was not remarkable in this series (Table 1).

The gold standard diagnostic method for the disease is DIF examination of perilesional skin which must demonstrate linear IgA deposition along the basement membrane zone as the disease name implies. Other immunoreactants such as IgG, IgM, and/or C3 may show an association with dominant IgA antibody deposition in DIF examination of LABD patients.^2^, ^13^ In a recent study, clinical manifestations and serological findings were analyzed in 101 patients with linear IgA deposition with or without linear C3 deposition along the basement membrane zone, excluding those with concurrent linear IgG and/or IgM deposition among LABD patients, based on DIF findings, unlike the present study.^9^ Although IgG deposition was not present along the basement membrane zone with DIF in this study population, approximately half of the patients (54 of 101 patients) had IgG anti-basement membrane zone antibodies detected either by IIF, immunoblotting, or ELISA. In addition, the patients with and without IgG antibodies detected either by IIF, immunoblotting, or ELISA were compared regarding clinical manifestations and no statistical differences were found in any of the variables between the two groups.^9^ In another study reported from Japan analyzing 213 cases previously reported in the literature, clinical appearance did not show any obvious difference between the IgA/G type and IgA type of LABD patients, and this finding was also confirmed in other large series.^13^, ^17^ However, it has also been stated that IgG contributed less frequently to the infantile type than to the adult type and most cases showing localization of antigen to the dermal side in IIF were the IgA/G type of LABD.^17^ It was suggested that there is no need to exclude the patients showing IgG deposition in addition to dominant IgA from a diagnosis of LABD.^9^, ^13^, ^17^ In the present series, IgG deposition on the basement membrane zone was detected in 35% of the patients in DIF examination, a ratio similar to that reported in other studies.^12^, ^13^, ^19^

The IIF has also been used to detect circulating antibodies against different antigens and its sensitivity ranges between 30% and 50% in LABD patients.^3^, ^6^ Circulating IgA antibodies revealed a positive result in the IIF examination in 33.3% of patients of whom the test could be performed, which was consistent with the literature.^3^, ^6^, ^12^

Cutaneous manifestations in LABD are heterogeneous and may mimic other bullous diseases. Descriptions in the literature range from target-like to erythematous papules, urticarial plaques, or vesiculobullous eruptions. Lesions may appear as tense arciform bullae in a “cluster of jewels” configuration as seen in bullous pemphigoid or less commonly as grouped papulovesicles as seen in dermatitis herpetiformis which were the clinical presentation mostly recorded in patients (Fig. 2B). Furthermore, clinical presentations with similarities to Stevens-Johnson syndrome, and toxic epidermal necrolysis have also been described previously which were not recorded in this series.^2^, ^3^, ^13^

LABD can also have mucosal involvement in about 40%‒80% of patients with a lower ratio in childhood LABD, as in this series.^2^, ^6^, ^12^, ^13^^,^^19^, ^20^ The most commonly involved mucosal surfaces are the oral mucosa and conjunctiva, which were detected in 34.6% and 3.8% of this series, respectively. Previously, it was shown that LABD patients with mucosal involvement are at risk of chronic evolution of their disease.^13^ Cutaneous lesions typically heal without scarring, but mucosal involvement may lead to scarring.^6^, ^8^, ^13^, ^21^ In one adult patient in this series with conjunctival involvement, a treatment-resistant disease course including cyclophosphamide, immunoadsorption, intravenous immunoglobulin, and rituximab was present, leading to significant conjunctival scarring and loss of visual acuity.

Systematic reviews with randomized clinical trials regarding treatment are scarce in some autoimmune bullous dermatoses including LABD due to the low incidence of these diseases.^22^ Various therapeutic options have been used for LABD since its original discovery in the 1970s, but the most commonly used one is oral dapsone worldwide.^2^, ^6^, ^12^ The most commonly used therapeutic option for LABD patients in this series was also oral dapsone with an adjunctive administration of oral corticosteroids to gain control over the disease in most of them. Interestingly, multiple antibiotic therapies including erythromycin and tetracyclines have also proven to be beneficial in controlling the disease without any underlying infectious etiology.^6^ Nicotinamide may be beneficial as an adjunctive treatment which was used in only two of the adult patients as a combination treatment with tetracyclines.^6^ It was not possible to conclude the treatment response in addition to the disease course of the child patients since the number was small.

The main limitations of the present study were its retrospective nature, to be conducted in a single tertiary center over a very long period and the small number which depends on the rarity of the disease. In addition, immunoblotting could not be performed in the studied cohort.

## Conclusion

LABD may occur at two separate peaks, one in the second and the other in the sixth decade of life with a female predominance, as in this series. Other immunoglobulins may be associated with dominant IgA antibody deposition in DIF examination of LABD patients. The most commonly used therapeutic option for LABD patients was oral dapsone with an adjunctive administration of oral corticosteroids in most of the studied patients.

## Financial support

None declared.

## Authors’ contributions

Tugba Atci: The study concept and design, data collection, or analysis and interpretation of data, statistical analysis, writing of the manuscript or critical review of important intellectual content, data collection, analysis and interpretation, effective participation in the research guidance, intellectual participation in the propaedeutic and/or therapeutic conduct of the studied cases, critical review of the literature, final approval of the final version of the manuscript.

Gizem Pehlivan Ulutas: Data collection, or analysis and interpretation of data, statistical analysis, writing of the manuscript or critical review of important intellectual content, data collection, analysis and interpretation, effective participation in the research guidance, intellectual participation in the propaedeutic and/or therapeutic conduct of the studied cases, critical review of the literature, final approval of the final version of the manuscript.

Ecem Güreler Sirkeci: Data collection, or analysis and interpretation of data, statistical analysis, writing of the manuscript or critical review of important intellectual content, data collection, analysis and interpretation, effective participation in the research guidance, intellectual participation in the propaedeutic and/or therapeutic conduct of the studied cases, critical review of the literature, final approval of the final version of the manuscript.

Rıfkiye Küçükoğlu: The study concept and design, data collection, or analysis and interpretation of data, statistical analysis, writing of the manuscript or critical review of important intellectual content, data collection, analysis and interpretation, effective participation in the research guidance, intellectual participation in the propaedeutic and/or therapeutic conduct of the studied cases, critical review of the literature, final approval of the final version of the manuscript.

## Conflicts of interest

None declared.
